# RECAP-seq: restriction enzyme-based CpG-methylated fragment amplification for early cancer detection

**DOI:** 10.1038/s41598-025-24708-y

**Published:** 2025-11-19

**Authors:** Dongju Shin, Taehoon Kim, Jaywon Lee, Hwang-Phill Kim, Tae-You Kim, Duhee Bang

**Affiliations:** 1 Department of Chemistry, Yonsei University, 50 Yonsei-ro, Seodaemun-gu, Seoul, 03722 Korea; 2 IMBdx Inc, Seoul, 08506 Republic of Korea; 3 Cancer Research Institute, Seoul National University, Seoul, 03080 Republic of Korea; 4 Department of Internal Medicine, Seoul National University Hospital, Seoul, 03080 Republic of Korea; 5 Department of Molecular Medicine and Biopharmaceutical Sciences, Graduate School of Convergence and Technology, Seoul National University, Seoul, 08826 Republic of Korea

**Keywords:** DNA methylation, DNA restriction enzymes, Enrichment, Detection, Cell-free DNA, Colorectal cancer, Biological techniques, Biomarkers, Cancer, Genetics, Molecular biology, Oncology

## Abstract

Aberrant DNA methylation drives cancer development, yet current screening methods require substantial resources for targeted enrichment across multiple CpG-rich regions. Early cancer detection in cell-free DNA (cfDNA) presents additional challenges due to low circulating tumor DNA fractions (0.01–10%) that dilute cancer-specific signals. To address these limitations, we developed Restriction Enzyme-based CpG-methylated fragment AmPlification sequencing (RECAP-seq) to selectively enrich hypermethylated fragments from existing Enzymatic Methyl-seq (EM-seq) libraries. RECAP-seq combines EM-seq library preparation with BstUI restriction enzyme digestion to target CGCG motifs, achieving preferential enrichment of CpG islands. With spike-in experiments using cell line mixtures, RECAP-seq successfully distinguished samples as low as 0.001%. The method identified 7,091 hypermethylated markers, including *ALX4* which showed progressive increases with colorectal cancer stage. Clinical validation using cfDNA from 35 healthy donors and 47 colorectal cancer patients demonstrated robust detection with an area under the curve (AUC) of 0.932, achieving 78.7% sensitivity at 95% specificity.

**Supplementary Information:**

The online version contains supplementary material available at 10.1038/s41598-025-24708-y.

## Introduction

Genome-wide aberrant DNA methylation is recognized as a fundamental epigenetic mechanism that drives cancer development^1–4^. Among these methylated regions, CpG islands represent critical regulatory elements, as these CpG- and GC-rich genomic regions, are predominantly localized at gene promoters^5–8^. Mechanistically, promoter CpG island methylation results in transcriptional silencing through diminished transcription factor binding affinity and recruitment of methyl-CpG binding domain proteins that associate with histone deacetylases^9–13^. Tumor suppressor genes are commonly inactivated through CpG island hypermethylation, thereby facilitating tumorigenesis^14–18^. Additionally, the CpG island methylator phenotype (CIMP) classification enables cancer subtype stratification based on extensive CpG island methylation patterns^19–21^. These observations collectively emphasize the pivotal role of CpG island methylation in cancer pathogenesis.

To identify cancer-associated methylation markers genome-wide, various sequencing-based methods have been applied. Whole-genome bisulfite sequencing (WGBS) and Enzymatic Methyl-seq (EM-seq) provide single-base resolution of methylation status^22,23^, but necessitate sequencing of the entire genome, which yields relatively low fraction of CpG-rich regions^24^. Additionally, these methods require substantial data volumes, extensive processing times, and considerable costs^25^, rendering them impractical for large-scale sample screening applications. Reduced representation bisulfite sequencing (RRBS) enriches CpG-dense regions through restriction enzyme digestion and size selection, thereby reducing sequencing requirements^26^. These approaches commonly employ CCGG-targeting enzyme MspI, which effectively target CpG-rich regions for this enrichment process^27^. However, RRBS presents challenges when applied to cell-free DNA (cfDNA) analysis because the naturally fragmented state of cfDNA precludes standard size selection protocols^28,29^, requiring specialized experimental design for library preparation^27,30^. Moreover, RRBS captures both methylated and unmethylated DNA fragments without bias, meaning that when methylated molecules represent only a small percentage of the total population (e.g., 0.1–1%), they may be overwhelmed by the unmethylated majority, limiting the method’s ability to detect clinically relevant low-abundance methylation markers. To address this issue, Methylated DNA immunoprecipitation sequencing (MeDIP-seq) can selectively enrich methylated fragments but lacks single-base resolution and provides limited control over the genomic regions that are sequenced^31,32^. Hybridization-based methylation panels, such as Agilent SureSelectXT Methyl-Seq, offer good control over the sequenced regions but the panel itself is expensive and drives up overall experimental costs^33^.

To address these limitations, we developed Restriction Enzyme-based CpG-methylated fragment AmPlification sequencing (RECAP-seq). RECAP-seq applies enzymatic DNA digestion at CGCG sites to existing EM-seq libraries, achieving dual selectivity: first, by enriching CpG islands through targeted restriction enzyme digestion, and second, by selectively capturing hypermethylated fragments within those CpG island regions. This approach specifically enriches cancer-associated CpG islands, where hypermethylation serves as a key cancer marker, enabling targeted sequencing of tumor-derived cfDNA. By concentrating sequencing efforts on methylated fragments, RECAP-seq significantly reduces required data volume while detecting low-abundance methylated DNA that would be missed in standard whole-genome approaches. Here, we demonstrate RECAP-seq’s capability to detect low-abundance cancer DNA in spike-in genomic mixtures (NA12878 and SW480) and validate its performance in cfDNA samples from 35 healthy individuals and 47 colorectal cancer patients.

## Results

### Overview of RECAP-seq

**Fig. 1:**
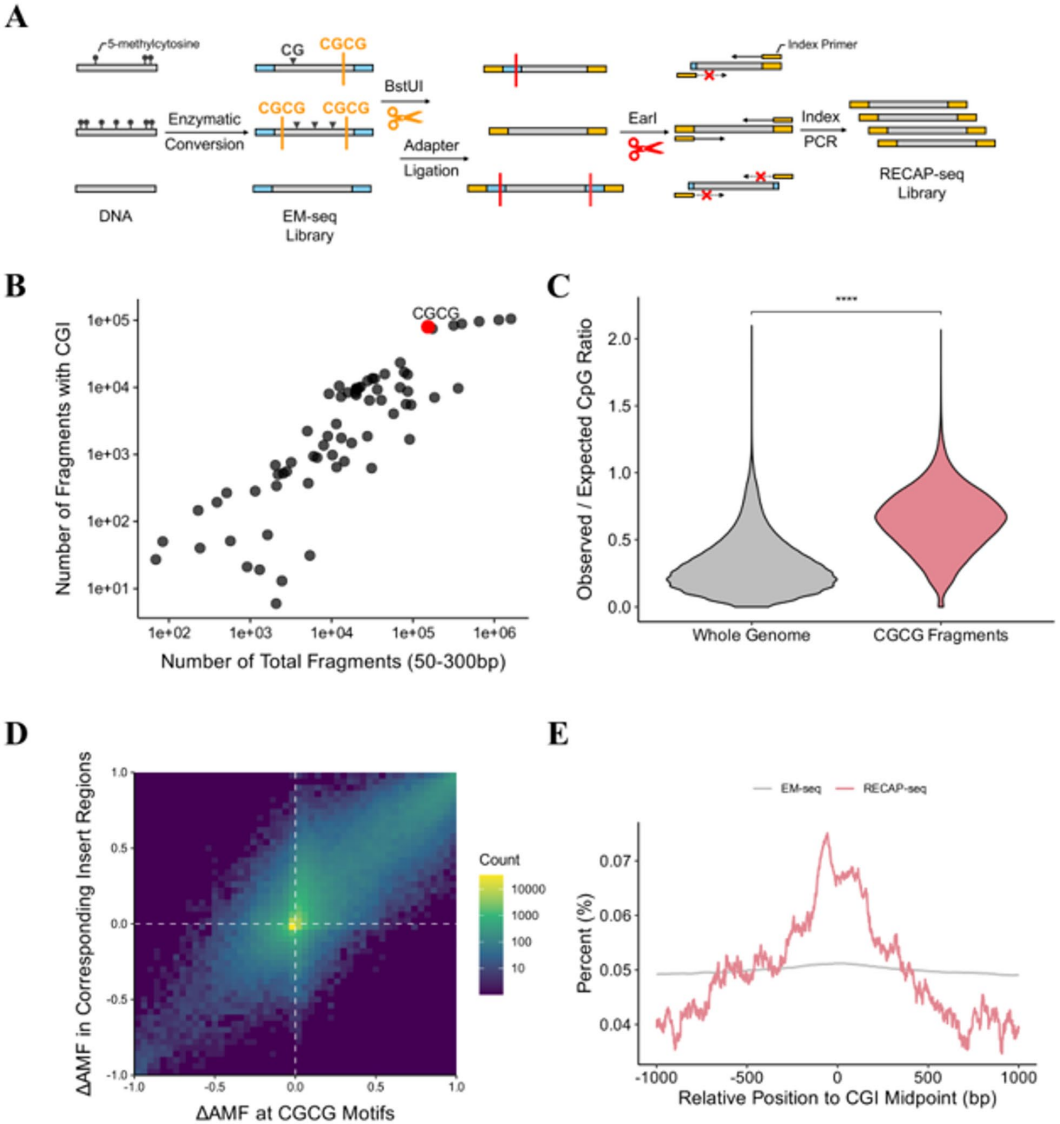
Restriction enzyme-based CpG-methylated fragment amplification—RECAP-seq. **(A)** Schematic of RECAP-seq experimental workflow. The EM-seq library (Truseq adapter) is treated with BstUI, and the resulting fragments are ligated to Nextera adapters. Next, EarI is used to remove any remaining Truseq adapter sequences. Index PCR is then performed, and fragments with Nextera adapters at both ends are selectively amplified. **(B)** Total number of fragments (50–300 bp) generated in the reference genome and the number of those fragments overlapping CpG islands. **(C)** Distribution of observed/expected CpG ratio in whole genome 300 bp windows and in CGCG fragments (50–300 bp). Regions shown here are filtered for GC content ≥ 50%. The observed/expected CpG ratio is calculated as the number of observed CpGs divided by the expected number of CpGs, estimated as (number of C × number of G)/region length^6^. **(D)** Genome-wide differential methylation (ΔAMF) at CGCG motifs and their corresponding insert regions in EM-seq data. Differential methylation was defined as AMF_SW480_ – AMF_NA12878_. For each CGCG fragment, differential methylation of two flanking CGCG motifs were averaged (Pearson *r* = 0.831, *n* = 143,485, *p* ≤ 0.001). **(E)** Average number of RECAP-seq NA12878 bases covering each relative position to the CpG island (CGI) midpoint, presented as percentages. Technical replicates were averaged at each position.

EM-seq is a bisulfite-free method for DNA methylation profiling that converts unmethylated cytosines to uracil, while leaving methylated CpGs unchanged. This results in libraries with few cytosines, most of which correspond to methylated CpG sites. RECAP-seq leverages this property by using a restriction enzyme that specifically recognizes and cuts CGCG motif, selectively fragmenting methylated, information-rich region in genome (Fig. 1A). These fragments are then ligated to new sequencing adapters and amplified, generating a refined library enriched for methylated CpG-containing sequences. This workflow can produce molecules with chimeric adapters, derived from fragments that are either uncut or cleaved at only one end. To remove these byproducts, EarI digestion step is applied and the following PCR step selectively amplifies fragments with adapters at both ends. To determine the optimal restriction enzyme for CpG-rich region enrichment, we performed in silico screening of 4–6 bp sequences containing CG across the reference genome (Materials and methods). This analysis identified CGCG, the target recognition site for BstUI, as overlapping most frequently with CpG islands and yielding the highest number of fragments within the size range of 50–300 bp (Fig. 1B). Hereafter, we define CGCG fragments as genomic regions flanked by CGCG motifs at fragment ends, filtered to 50–300 bp inserts. CGCG fragments exhibit significantly higher observed/expected CpG ratios compared to whole-genome windows (both filtered for GC contents ≥ 50%), confirming that CGCG fragments are CpG-rich (Fig. 1C). Coverage analysis of CGCG fragments also demonstrates targeted enrichment of CpG islands at the genomic region level (Fig. S1).

Previous studies have demonstrated that DNA methyltransferases methylate adjacent CpG sites in a processive manner during a single DNA-binding event^34–36^, implying that once de novo methylation occurs, CpG islands may be further methylated across neighboring sites without enzyme dissociation. To test whether we can enrich continuously hypermethylated regions by cleaving CGCG sites in an EM-seq library, we calculated the average methylation fraction (AMF) within the flanking CGCG motifs and their corresponding insert regions (the sequence between CGCG motifs) using EM-seq data from NA12878 (normal) and SW480 (colorectal cancer) genomic DNA (gDNA) (Fig. 1D). We defined the differential methylation as AMF_SW480_ – AMF_NA12878_, reasoning that higher differential methylation increases the probability that CGCG motifs in SW480 are fully methylated and thus more likely to be cleaved by BstUI. Indeed, differential methylation values at CGCG motifs and insert regions were strongly correlated (Pearson *r* = 0.831, *p* ≤ 0.001), indicating that dual-end restriction effectively targets successively hypermethylated fragments. RECAP-seq libraries captured over 80% of fragments containing both CGCG motifs (Fig. S2), confirming efficient recovery of CGCG fragments. Given CGCG fragments’ strong association with CpG islands (Fig. S1), RECAP-seq is expected to preferentially target these regions. Indeed, RECAP-seq coverage peaked sharply at CpG island midpoints in NA12878 gDNA, whereas EM-seq coverage was broadly distributed genome-wide (Fig. 1E). Together, these data demonstrate that RECAP-seq selectively enriches CpG-rich, hypermethylated regions.

### RECAP-seq maintains concordance with EM‑seq

**Fig. 2:**
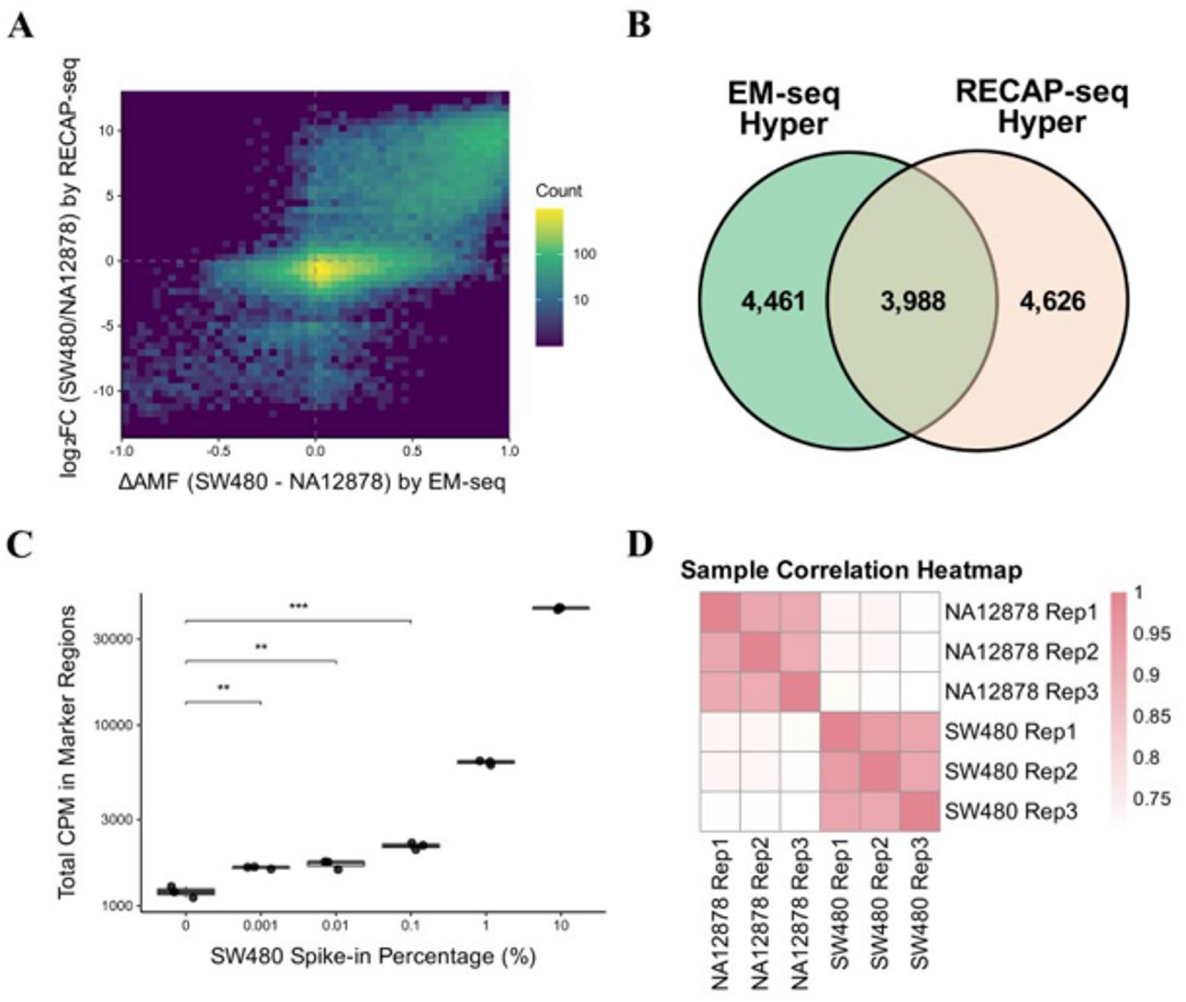
Analytical validation of RECAP-seq demonstrates reproducibility and sensitivity in detecting low spike-in samples. (**A)** Correlation of differential methylation signals by EM-seq and RECAP-seq. Scatterplot comparing ΔAMF from EM-seq with log₂FC from RECAP-seq. FC refers to the normalized count of SW480 divided by NA12878 (Pearson *r* = 0.733, *n* = 35,418, *p* ≤ 0.001). (**B)** Overlap of EM-seq hypermethylated markers and RECAP-seq hypermethylated markers, shown as Venn diagram. The significance of the overlap was assessed by hypergeometric test (*p* ≤ 0.001). (**C)** Total CPM in spike-in hypermethylated marker regions for NA12878-SW480 in vitro mixtures at increasing SW480 spike-in percentages (8,614 marker regions selected from RECAP-seq data; *n* = 3 per group). (**D)** Heatmap of Spearman correlation between RECAP-seq triplicates.

Given the method’s design to target fragments with fully methylated CGCG motifs at both ends, we expect that individual captured fragments would exhibit predominantly hypermethylated profiles. To confirm this, we examined read-level methylation values, calculated as the fraction of methylated CpGs relative to the total number of CpGs within each read. As anticipated, read-level methylation values were skewed toward higher levels compared to EM-seq (Fig. S3). This enrichment bias necessitates interpreting RECAP-seq data as counts of captured CGCG fragments rather than AMFs. To evaluate quantitative concordance with EM-seq, we compared RECAP-seq log₂ fold changes (FC) in CGCG fragment counts (SW480 vs. NA12878) to corresponding EM-seq differential methylation values. We observed a significant positive correlation (Pearson *r* = 0.733, *p* ≤ 0.001; Fig. 2A), demonstrating that the enrichment bias does not compromise the method’s ability to capture biologically relevant methylation differences.

### Analytical validation of RECAP-seq with spike-in controls

To assess RECAP-seq’s potential for clinical diagnostics, we performed spike-in experiments by mixing SW480 gDNA into NA12878 gDNA at various ratios, with both DNA samples sheared to ~ 180 bp to resemble cfDNA size. Since SW480 is derived from colorectal cancer cells while NA12878 represents healthy control, this experimental design simulates the detection of circulating tumor DNA from patient samples in vitro. We tested SW480 fractions ranging from 0.001% to 10% to evaluate RECAP-seq’s ability to distinguish cancer-associated methylation signals.

To identify genomic regions that best represent SW480-derived signals for spike-in detection, we performed marker selection on CGCG fragments from unmixed NA12878 and SW480 samples (Materials and methods). Correlation analysis between normalized read counts and spike-in fractions revealed that hypermethylated CGCG fragments exhibited higher correlation values compared to hypomethylated regions (Fig. S4). This means that the number of hypermethylated CGCG fragments increases as the spike-in fraction increases, while the hypomethylated CGCG fragments did not show this pattern clearly. Given that RECAP-seq is inherently designed as a method for capturing hypermethylated fragments, and considering the suboptimal performance observed with hypomethylated markers in the experimental dataset, we selected hypermethylated markers for subsequent analyses. Hypermethylated RECAP-seq markers shared overlap with 47.2% of EM-seq hypermethylated markers (Fig. 2B). To evaluate the performance of RECAP-seq, we calculated counts per million (CPM)-normalized counts for each marker region to account for differences in library size across samples. CPM-normalized counts within each marker region were summed to calculate total CPM in marker regions. The analysis demonstrated that RECAP-seq successfully distinguished samples with low SW480 spike-in fractions as low as 0.001% (Fig. 2C), indicating the high sensitivity for detecting minimal levels of hypermethylated DNA with consistent reproducibility across technical replicates (Fig. 2D, Fig. S5).

### Validation of RECAP-seq by colorectal cancer CfDNA samples

**Fig. 3:**
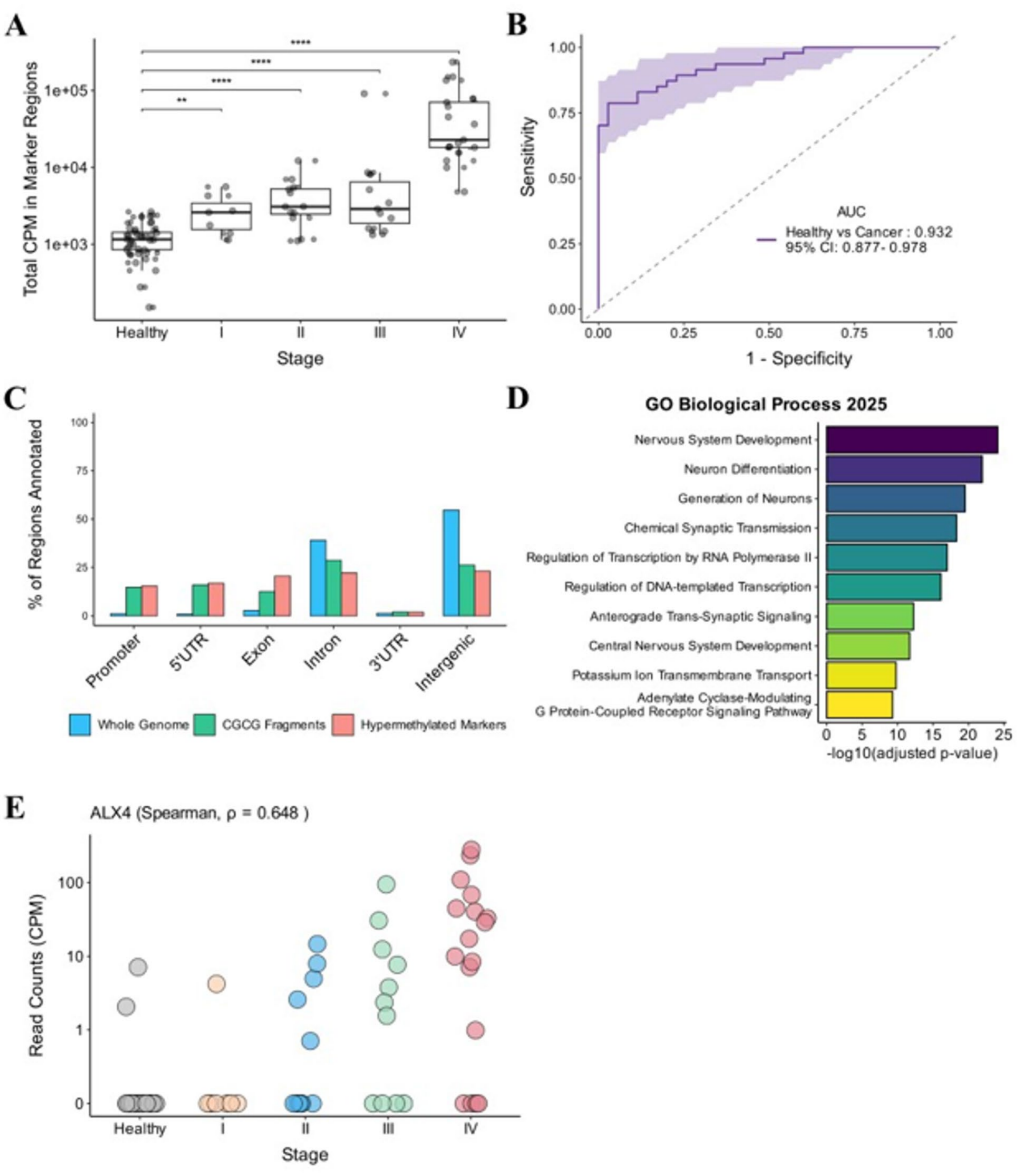
Clinical validation of RECAP-seq cfDNA markers for distinguishing healthy controls from colorectal cancer. (**A)** Total CPM in clinical samples’ hypermethylated marker regions grouped by cancer stage (7,091 markers; healthy, *n* = 35; Stage I, *n* = 7; Stage II, *n* = 12; Stage III, *n* = 11; Stage IV, *n* = 17; total, *n* = 82). (**B)** Receiver operating characteristic (ROC) curve for the validation cohort (healthy, *n* = 35; cancer, *n* = 47) using total CPM across marker regions for classification. The ROC curve and AUC confidence interval were calculated by bootstrapping (*n* = 1,000). (**C)** Genomic annotation of whole genome 300 bp windows, CGCG fragments, and hypermethylated markers (whole genome: 9,603,454 regions; CGCG fragments: 153,261 regions; hypermethylated markers: 7,091 regions). (**D)** Pathway analysis of 2,093 unique genes overlapping hypermethylated markers. (**E)** CPM-normalized read counts for *ALX4* plotted by cancer stage. Spearman correlation between CPM and stage is shown (healthy, *n* = 35; Stage I, *n* = 7; Stage II, *n* = 12; Stage III, *n* = 11; Stage IV, *n* = 17; total, *n* = 82).

To evaluate RECAP-seq’s potential for noninvasive cancer detection, we first identified hypermethylated markers by comparing RECAP-seq profiles of healthy cfDNA (*n* = 8) and colorectal cancer tissue samples (*n* = 8), yielding 7,091 markers (Materials and methods). We then calculated the sum of CPM-normalized read counts across these markers in the cfDNA cohort to distinguish healthy donors (*n* = 35) from cancer patients (*n* = 47) (Fig. 3A). RECAP-seq robustly differentiated healthy cfDNA from cancer cfDNA (*p* ≤ 0.01 for each stage comparison) without gender confounding (*p* > 0.05, Fig. S6). Receiver operating characteristic (ROC) analysis yielded an area under the curve (AUC) of 0.932 (95% CI: 0.877–0.978) for cancer versus healthy samples (Fig. 3B), with 78.7% sensitivity at 95% specificity. For early-stage cases (stages I and II), sensitivity was 68.4% at 95% specificity. Importantly, when applying a CPM > 5 cutoff, an average of 1028.4 hypermethylated markers were detected in cancer cfDNA samples, whereas only 71.5 markers were detected in healthy cfDNA samples (Table S1). From the full CGCG fragment pool, we successfully identified hypermethylated markers that more accurately reflect cancer progression (Fig. S7), confirming that our selection identifies cancer-associated signals. These results demonstrate RECAP-seq’s clinical potential in colorectal cancer screening.

We further investigated the biological relevance and generalizability of the 7,091 cfDNA-derived hypermethylated markers through genomic feature and CpG context annotations. Clinical cfDNA markers were enriched in exonic regions and CpG islands relative to both whole-genome and CGCG fragment baselines (Fig. 3C, Fig. S8), with similar enrichment patterns observed for spike-in sample markers (Fig. S9A, B). Pathway analysis revealed significant overrepresentation of genes involved in nervous system development (Fig. 3D, Fig. S9C). Previous studies have established associations between neuronal pathways and colorectal cancer progression^37–41^, indicating that hypermethylation of these genes may promote cancer development. Among the identified markers, *ALX4* demonstrated progressively increased signal with cancer stage (Fig. 3E) and exhibited robust support from cohort analyses^42–46^. Notably, *ALX4* functions as a tumor suppressor whose silencing by promoter hypermethylation contributes to tumorigenesis^47^. Additional markers relevant to colorectal cancer, including *NPY*^48–50^, *ITGA4*^51–53^, and *IRF4*^54–56^, also exhibited progressive increases with cancer stage (Fig. S10, Table S2). Collectively, these findings indicate that RECAP-seq can facilitate cancer detection using markers with both biological significance and clinical utility.

## Discussion

RECAP-seq selectively enriches CpG-rich, hypermethylated regions through restriction enzyme digestion of EM-seq libraries. This strategy concentrates sequencing depth on CpG islands, which are frequent sites of cancer-associated hypermethylation. It enables efficient marker discovery with high regional specificity and reduced data burden, without relying on costly methylation capture panels. In addition, the unused portion of the EM-seq library remains compatible with whole-genome methylation analysis if needed. We demonstrated that RECAP-seq reliably detects low-fraction methylated DNA in cell line mixtures and distinguishes colorectal cancer cfDNA from healthy cfDNA with an AUC of 0.932. Notably, RECAP-seq achieved comparable or higher sensitivity than reported SEPT9-based assays while maintaining similar specificity levels^57,58^. Taken together, these features position RECAP-seq as a platform for large-scale cancer screening.

Nonetheless, RECAP-seq has several inherent limitations. First, RECAP-seq captures hypermethylated fragments while missing hypomethylated regions, which are also important in colorectal cancer pathophysiology. Second, this selective capture does not accurately quantify methylation at individual CpG loci, as it primarily detects fully methylated CGCG fragments while underrepresenting intermediate methylation levels. This bias toward highly methylated regions may restrict the identification of markers that distinguish between low from intermediate methylation states. Third, enzyme efficiency limits capture. BstUI does not cleave all fully methylated CGCG sites, necessitating size selection, and EarI digestion of chimeric adapters is incomplete (Fig. S2). Further optimization of enzyme reactions and enhanced cleavage efficiency will be required to broaden RECAP-seq’s applicability. Fourth, the enrichment of CpG-rich sequences inherent to the RECAP-seq methodology reduces nucleotide diversity in sequencing libraries, which might reduce base quality and increase the proportion of unmapped reads compared to EM-seq (Fig. S11). Additionally, in samples with available demographic data, our cancer cohort had a higher median age than the healthy cohort (Table S3, Fig. S6). This age imbalance may confound our classification results and warrants validation with age-matched cohorts.

Future work should address two key areas. First, expanding the restriction-enzyme repertoire beyond BstUI could enable targeted enrichment of diverse methylation contexts from whole-genome methylation libraries. Different enzymes recognize distinct sequence motifs and methylation patterns, potentially broadening the scope of detectable markers. In silico screening and empirical validation of alternative enzymes will be essential^59^. Second, integrating machine-learning approaches into RECAP-seq analysis could enhance classification performance. Such algorithms could identify complex methylation patterns that are not readily apparent through conventional statistical methods^60^. Increasing sample size will facilitate the development of robust cfDNA-based classifiers built on RECAP-seq data.

## Conclusion

Aberrant DNA methylation in CpG islands is a common epigenetic alteration in cancer, yet screening multiple CpG-rich regions and detecting low cfDNA signals requires substantial resources. RECAP-seq addresses these challenges by coupling EM-seq library preparation with restriction enzyme digestion to enrich CpG-dense, hypermethylated fragments. We demonstrated its utility in both cell line mixtures and clinical cfDNA samples, achieving sensitive detection of cancer-associated methylation patterns without complex bioinformatic models. By enhancing regional and methylation selectivity while reducing sequencing requirements, RECAP-seq provides a practical platform for cancer screening applications. These findings establish RECAP-seq as a valuable tool that will accelerate translational research in cancer diagnostics.

## Materials and methods

### Clinical samples

We obtained plasma and matched tumor tissue from colorectal cancer patients, as well as plasma from healthy donors. In total, 45 blood samples (22 colorectal cancer, 23 healthy) and eight matched cancer tissue samples were collected. Plasma was separated by density centrifugation using Ficoll-Paque PLUS (GE Healthcare), and cfDNA was extracted with the QIAamp Circulating Nucleic Acid Kit (QIAGEN) according to the manufacturer’s instructions. An independent set of 58 cfDNA EM-seq libraries, 28 from colorectal cancer patients and 30 from healthy subjects, was obtained to evaluate RECAP-seq performance. All samples were collected under Institutional Review Board (IRB) approval from Seoul National University Hospital (IRB number H-2201-128-1294, H-1708-031-875). Informed consent was obtained from all participants and/or their legal guardians. All methods were performed in accordance with relevant guidelines and regulations.

### Cell line culture and gDNA preparation

gDNA for NA12878 was purchased from the Coriell Institute. SW480 colorectal adenocarcinoma cells (Korean Cell Line Bank) were cultured in RPMI 1640 (Gibco, USA) supplemented with 10% fetal bovine serum and 1% penicillin–streptomycin. gDNA was extracted using the DNeasy Blood & Tissue Kit (QIAGEN) according to the manufacturer’s instructions. Extracted gDNA was sheared to the average size of 180 bp using M220 Focused-Ultrasonicator (Covaris).

### NA12878 EM-seq data

Published NA12878 EM-seq data were obtained from European Nucleotide Archive (ENA) database (accession numbers: SRR20318435–SRR20318442). Four sequencing runs from each of two technical replicates were merged separately for the analysis.

### In silico restriction enzyme screening

We screened all 4–6 bp sequences containing CG to identify the optimal motif for CpG island enrichment. First, we generated 752 unique candidate motifs, then cross-referenced these against commercially available enzymes from the REBASE database (withrefm ver.504)^61^, yielding 69 supported motifs. For each motif and its reverse complement, we scanned the hg19 reference genome to identify all in silico fragments flanked by that motif, then defined insert regions as the sequence between the two cut sites. Inserts of 50–300 bp were retained to match practical library-size constraints and ensure mappability. We characterized the overlap between CGCG fragments and annotated CpG islands, which showed both high CpG island enrichment and genome-wide coverage (Fig. 1B). For sequencing data analyses, we filtered out any CGCG fragments overlapping ENCODE blacklist regions to avoid alignment artifacts^62^.

### RECAP-seq library preparation workflow

EM-seq libraries were constructed using the NEBNext Enzymatic Methyl-seq Kit (New England Biolabs) with TruSeq adapters, following the manufacturer’s protocol. 20 ng of EM-seq libraries were amplified by PCR to obtain sufficient substrate for subsequent restriction enzyme digestion. PCR reactions comprised 25 µL of 2X KAPA master mix, 2.5 µL each of P5 and P7-targeting primers (10 µM, Table S4), and nuclease-free water filled to total volume of 50 µL. 2X KAPA master mix was prepared with KAPA HiFi HotStart PCR Kit (KAPA Biosystems) following the manufacturer’s instructions. Amplification was performed in a thermal cycler under the following conditions: initial denaturation at 98 °C for 3 min; 10 cycles of 98 °C for 30 s, 60 °C for 30 s, and 72 °C for 1 min; and a final extension at 72 °C for 1 min. 1,000 ng of amplified EM-seq library was subjected to enzymatic digestion with 0.5 µL of BstUI (New England Biolabs), 2.5 µL of rCutSmart buffer (New England Biolabs), and nuclease-free water filled to total volume of 25 µL. Reactions were incubated at 60 °C for 1 h. Size selection of digested fragments was performed using a 1X volume of AMPure XP beads (Beckman Coulter) at room temperature for 1 h, and the supernatant was further purified with an Oligo Clean & Concentrator (Zymo Research), eluting in 25 µL of nuclease-free water.

Fragment ends were repaired and A-tailed by adding 3.5 µL of NEBNext Ultra II End Prep Reaction Buffer and 1.5 µL of NEBNext Ultra II End Prep Enzyme Mix (New England Biolabs), followed by incubation at 20 °C for 30 min and 65 °C for 30 min. Adapter ligation was carried out by adding 15 µL of NEBNext Ultra II Ligation Master Mix, 0.5 µL of NEBNext Ligation Enhancer, and 1.25 µL of Nextera adapter (0.6 µM), then incubating at 20 °C for 15 min. For clinical samples, an additional incubation at 45 °C for 30 min and 50 °C for 30 min was performed to maximize ligation efficiency^63^. Ligation products were purified with 2X AMPure XP beads and eluted in 35 µL of nuclease-free water. To remove residual TruSeq adapter sequences, 1 µL of EarI (New England Biolabs) and 4 µL of rCutSmart buffer were added to the purified ligation products, and the mixture was incubated at 37 °C for 1 h. The reaction was then purified with 2X AMPure XP beads and eluted in 20 µL of nuclease-free water.

Index PCR was performed in a 50 µL reaction containing 25 µL of 2X KAPA master mix and 2.5 µL each of forward and reverse index primers (10 µM). Thermal cycling conditions were as follows: initial denaturation at 98 °C for 3 min; 13 cycles of 98 °C for 30 s, 60 °C for 30 s, and 72 °C for 1 min; and final extension at 72 °C for 1 min. Indexed libraries were purified using 1.2X AMPure XP beads and eluted in 30 µL of nuclease-free water. Final library quantification was assessed by D1000 ScreenTape (Agilent) and were sequenced on an NovaSeq 6000 platform (Illumina) with 150 bp paired-end read out.

### Sequencing data processing

For RECAP-seq data, adapter trimming was performed on raw FASTQ files with FASTP (ver. 0.20.1)^64^, followed by removal of 1 bp from each end to eliminate any remaining adapter sequences. The alignment was performed with Bismark (ver. 0.24.2)^65^ on hg19 with the –non_directional parameter. Reads were then filtered to exclude those containing: (1) at least one non-converted base (using Bismark filter_non_conversion module), (2) mismatches in the restriction enzyme’s target sequence (CGCG), and (3) uncut sequences containing the CGCG motif within the read. The reads with CGCG fragments were counted using the bedtools (ver. 2.30.0)^66^ multicov option. RECAP-seq samples covering fewer than 2,000 unique CGCG fragments were removed from the analysis.

For EM-seq data, adapter trimming was performed on raw FASTQ files with FASTP, followed by alignment with Bismark on hg19 using the –non_directional parameter. Reads were then processed to exclude: (1) those containing at least one non-converted base (using Bismark filter_non_conversion module) and (2) duplicate reads (removed by GATK MarkDuplicates ver. 4.0.5.1)^67^. MethylDackel (ver. 0.3.0)^68^ was used to extract and merge CpG information on both strands within reads. The reads within CGCG fragments were counted using the bedtools multicov option. Sample information and sequenced read counts are provided in Table S3.

### Marker selection

To select RECAP-seq hypermethylated markers, count normalization and exactTest() were run in edgeR (ver. 3.30.3)^69^ to calculate log₂FC and false discovery rates (FDR). Thresholds of log₂FC > 5 and FDR < 0.01 were applied to identify marker regions. For the spike-in samples, we used triplicates of NA12878 and SW480. For the clinical samples, we used eight healthy cfDNA samples and eight colorectal cancer tissues. The colorectal cancer tissue data were generated in two technical replicates, so we took the average value of each CGCG-fragment region. The resulting marker selection yielded 8,614 markers for spike-in samples and 7,091 markers for clinical samples.

To select EM-seq-derived markers for evaluating RECAP-seq performance in spike-in samples, we calculated the AMF, defined as the total counts of methylated CpGs within the region divided by the total counts of methylated + unmethylated CpGs. EM-seq hypermethylated markers were defined as AMF < 0.2 in NA12878 and AMF > 0.8 in SW480. This yielded 8,449 markers.

### Annotation and pathway analysis

R package annotatr (ver.1.14.0)^70^ and GenomicRanges (ver.1.42.0)^71^ were used for genomic and CpG annotations. Unique genes identified in the markers were put in to Enrichr^72^ to find the relevant pathway.

### Statistical analysis

The asterisks represent ranges of p-values as follows: *p* > 0.05, ns; *p* ≤ 0.05, *; *p* ≤ 0.01, **; *p* ≤ 0.001, ***; and *p* ≤ 0.0001, ****. The asterisks shown indicate representative comparisons, and all pairwise p-values were adjusted using the FDR method^73^. In our study, Student’s t-tests were used to compare mean values, except for Fig. 3A and Fig. S6, which used the Wilcoxon rank-sum test. Boxplots show the interquartile range (Q1, median [Q2], and Q3). For correlation analysis, Pearson’s and Spearman’s correlation coefficients were calculated.

## Supplementary Information

Below is the link to the electronic supplementary material.

Supplementary Material 1

Supplementary Material 2

Supplementary Material 3

Supplementary Material 4

Supplementary Material 5

## Data Availability

Published NA12878 EM-seq data were obtained from the European Nucleotide Archive (ENA) database under accession numbers (SRR20318435–SRR20318442). RECAP-seq and EM-seq data generated in this study are available upon reasonable request from the corresponding author (D.B.).
