# Supplementary material for: RECAP-seq: restriction enzyme-based CpG-methylated fragment amplification for early cancer detection
Source: Sci Rep. 2025 Nov 19;15:40892. doi: 10.1038/s41598-025-24708-y (PMC12630582; doi:10.1038/s41598-025-24708-y)
Supplement: Supplementary file 5 — Supplementary Material 5 [file 41598_2025_24708_MOESM5_ESM.docx]

**Supplementary information**

**Fig. S1** Percentage of fragments spanning each position relative to the CpG island midpoint, assessed at the genomic region level. Random fragments were sampled from hg19 with the same length distributions as CGCG fragments (100 sampling iterations).


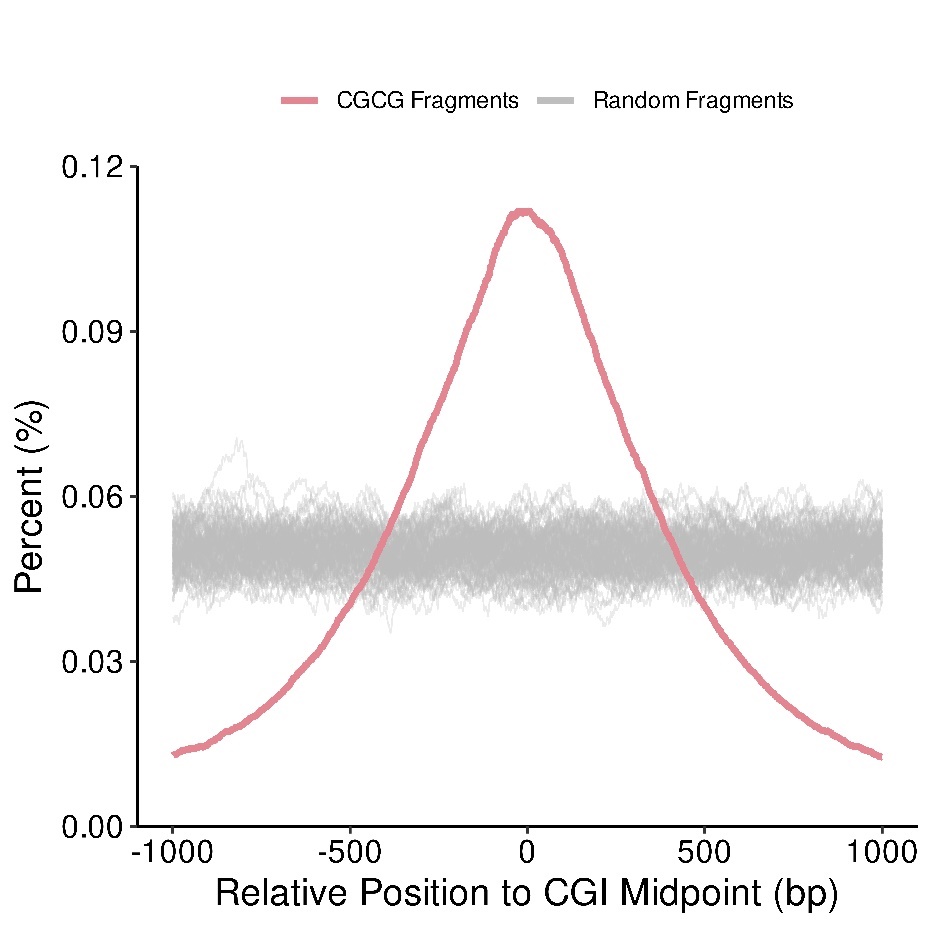


**Fig. S2** Percentage of mapped fragments cut by BstUI within each sample (*n* = 24). The x-axis indicates the number of CGCG motifs at both ends of paired-end reads.

**
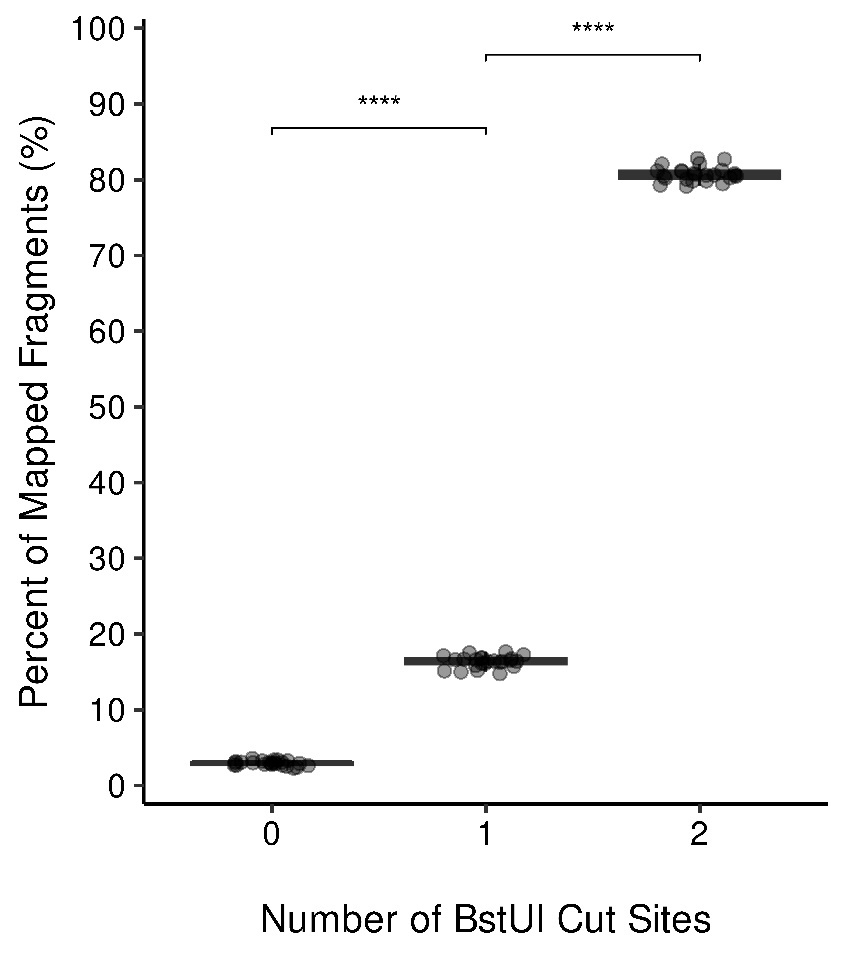
**

**Fig. S3** Comparison of read-level methylation values between EM-seq and RECAP-seq in NA12878. Read-level methylation values were calculated as the fraction of methylated CpGs relative to the total number of CpGs within each read, based on Bismark methylation calls.

**
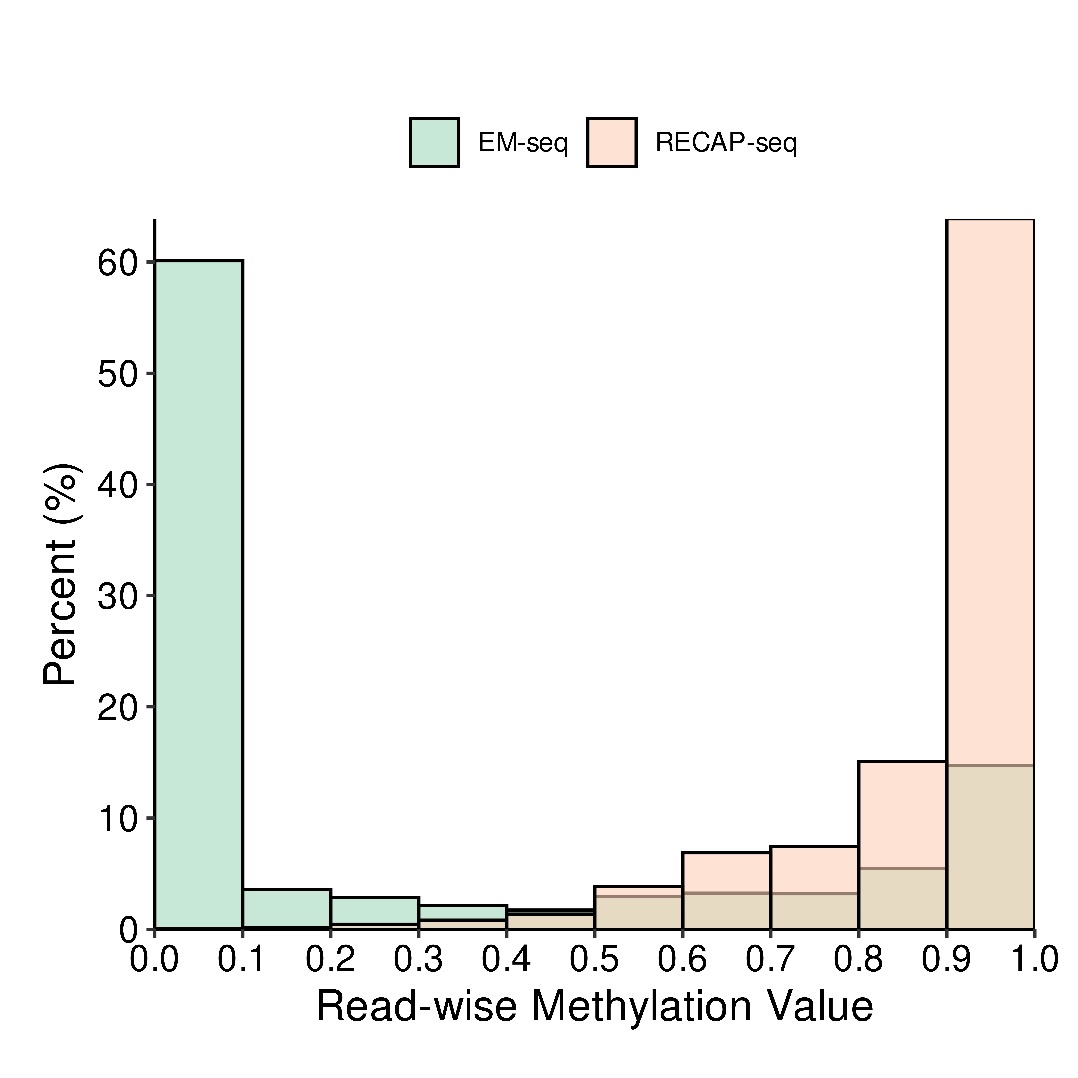
**

**Fig. S4** Absolute Spearman correlations for all CGCG fragments, hypermethylated markers, and hypomethylated markers in spike-in samples.

**
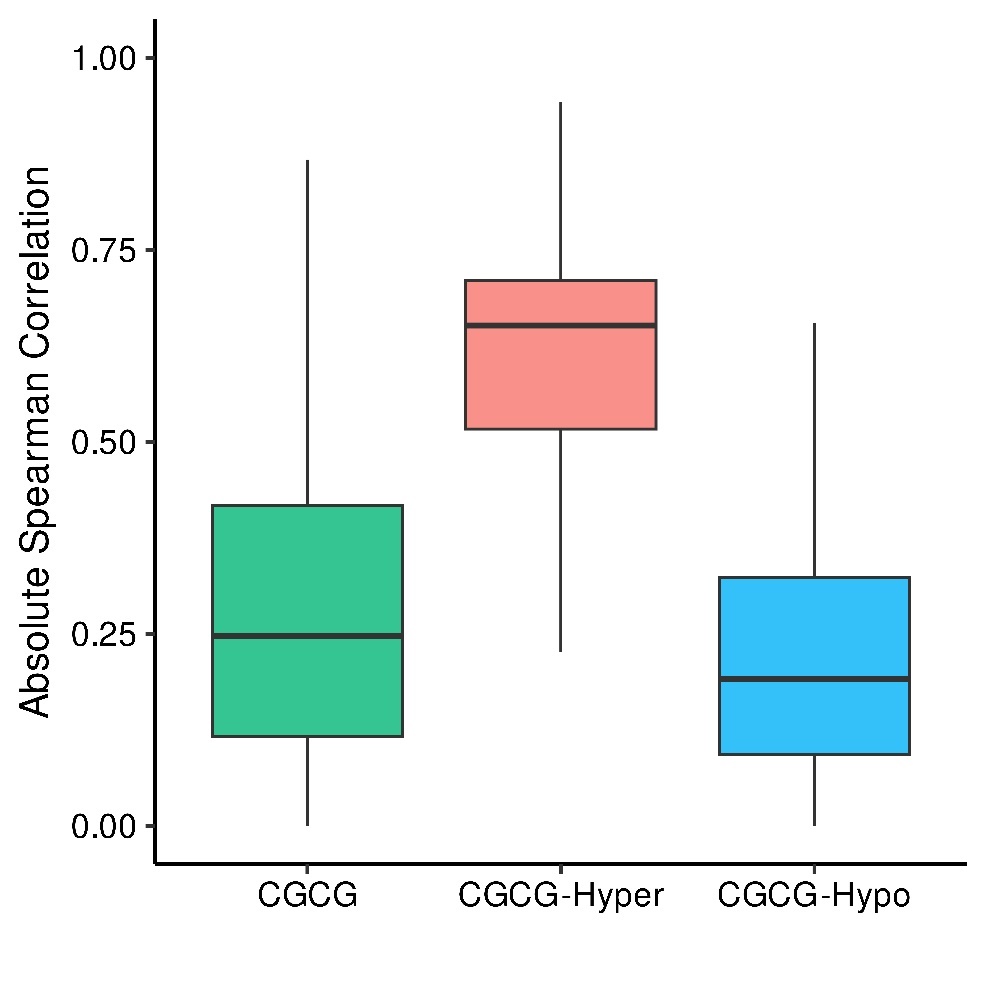
**

**Fig. S5** Replicate correlation within RECAP-seq. Pearson correlation is shown.

**
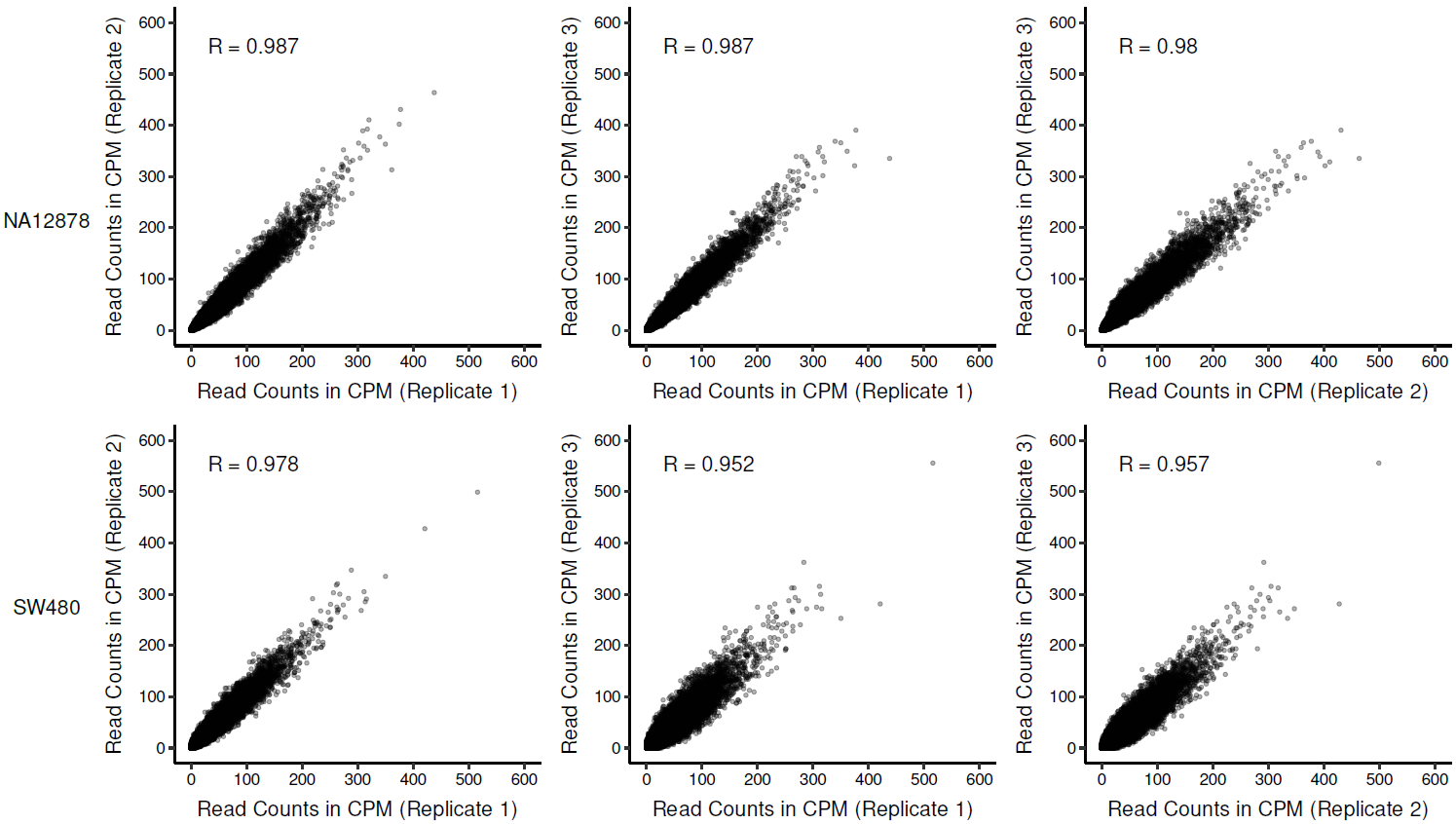
**

**Fig. S6** Analysis of potential confounding factors in RECAP-seq. Boxplots showing total CPM-normalized read counts across 7,091 hypermethylated marker regions in samples with available demographic data. **A** Comparison between female (*n* = 17) and male (*n* = 17) samples showed no significant difference (*p* > 0.05, Wilcoxon rank-sum test). **B** Comparison between samples from donors under 50 years (*n* = 17) and 50 years or older (*n* = 17) revealed a significant difference (*p* = 0.0054, Wilcoxon rank-sum test).

**
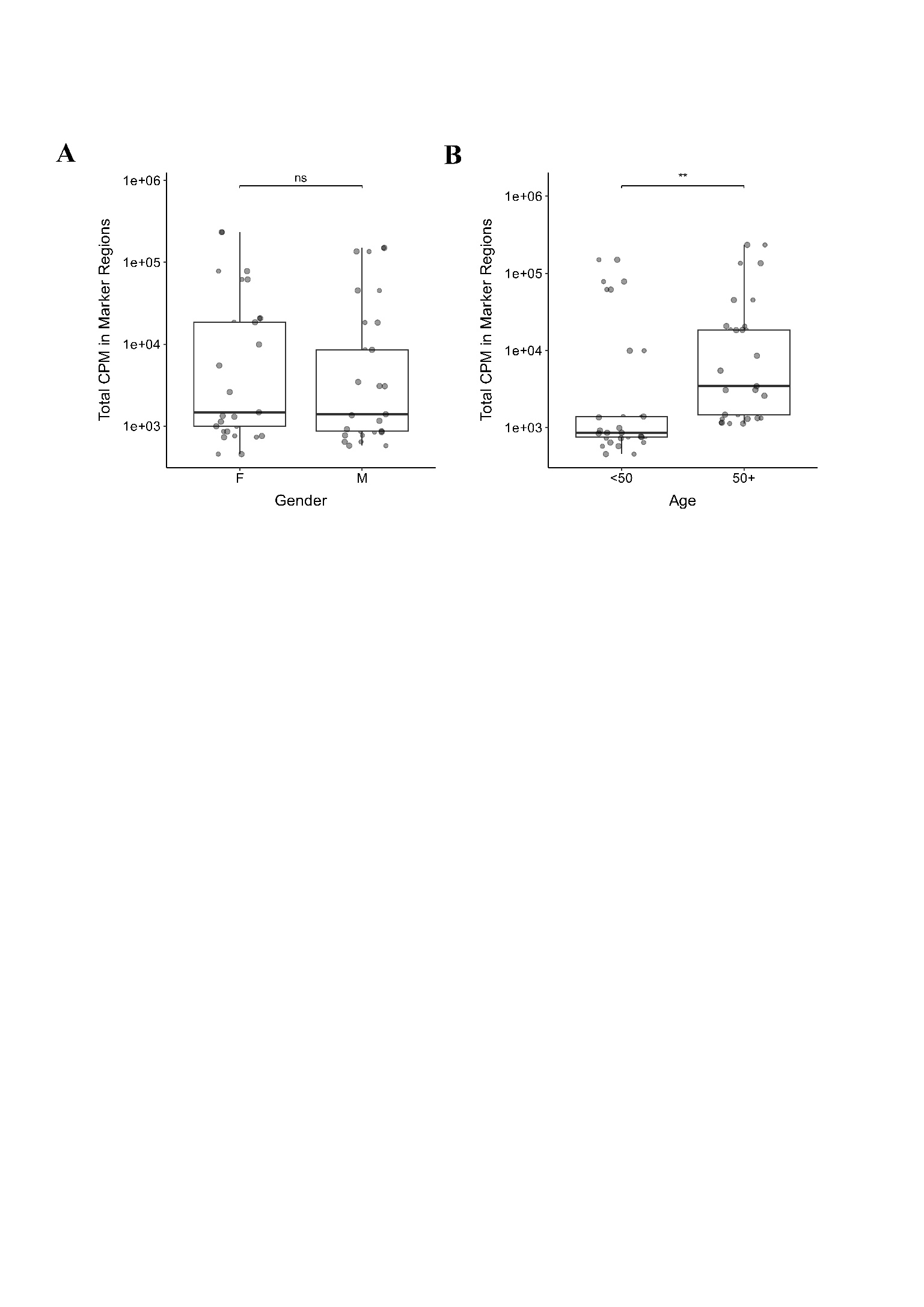
**

**Fig. S7** Absolute Spearman correlation between CPM-normalized counts and cancer stage progression for all CGCG fragments versus selected hypermethylated markers in clinical cfDNA samples (CGCG fragments, *n* = 58,126 out of 148,191 total fragments; fragments without calculable correlation were excluded; hypermethylated markers, *n* = 7,091). Higher correlations indicate that individual region’s CPM-normalized counts increase more consistently with advancing cancer stage.

**
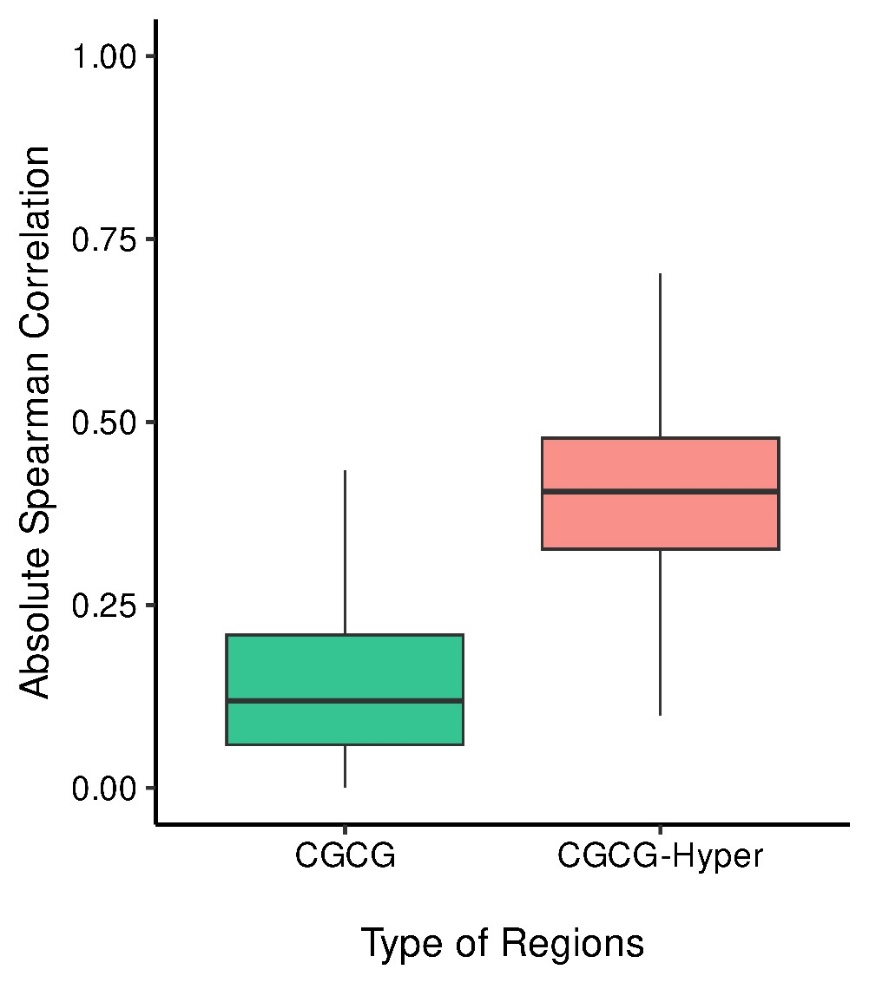
**

**Fig. S8** CpG annotation of clinical hypermethylated markers. (whole genome: 9,603,454 regions; CGCG fragments: 153,261 regions; hypermethylated markers: 7,091 regions)

**
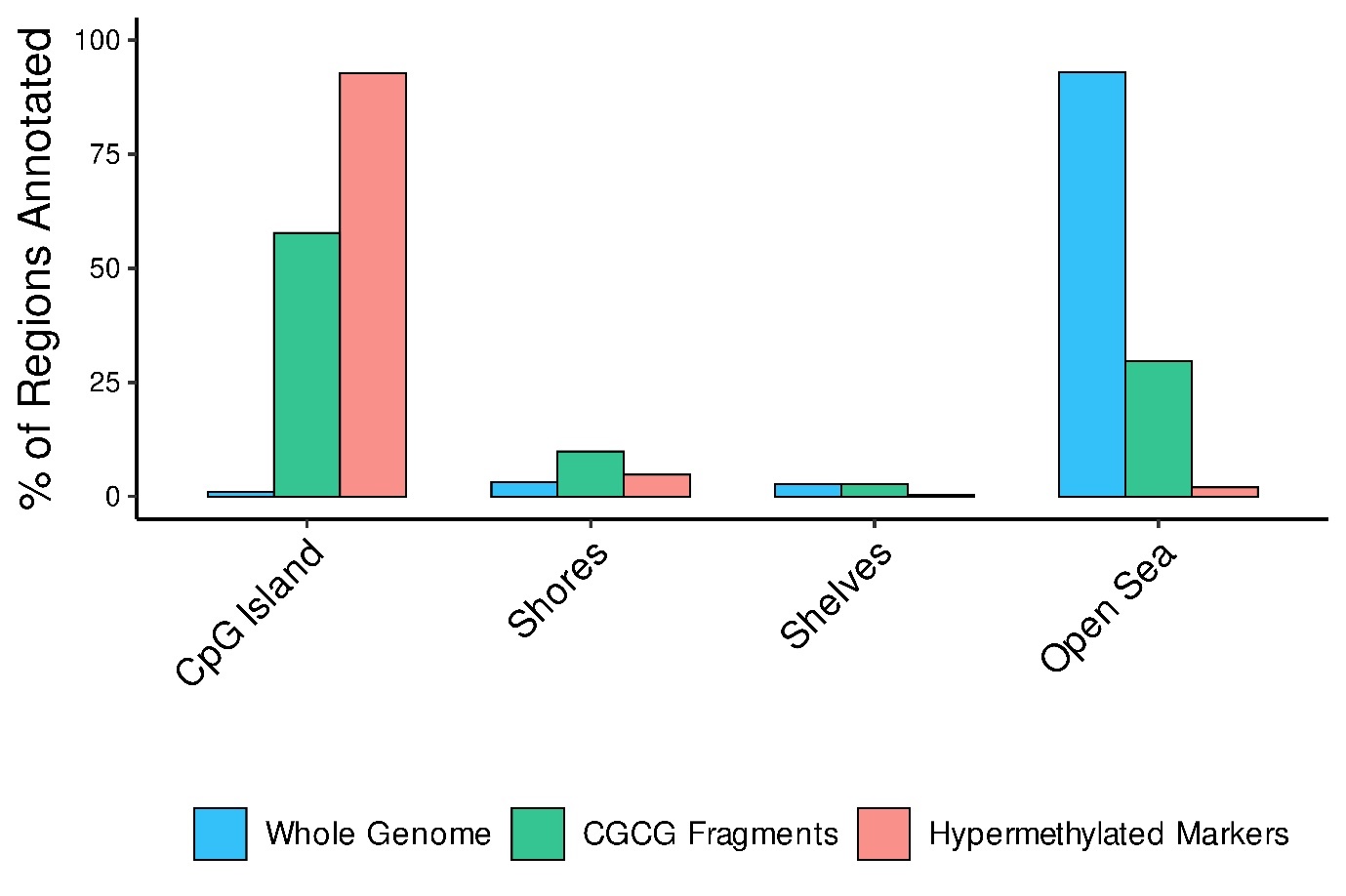
**

**Fig. S9 A, B** Genomic and CpG annotation of spike-in hypermethylated markers (whole genome: 9,603,454 regions; CGCG fragments: 153,261 regions; hypermethylated markers: 8,614 regions). **C** Pathway analysis of hypermethylated markers (2,800 unique genes input to Enrichr).

**
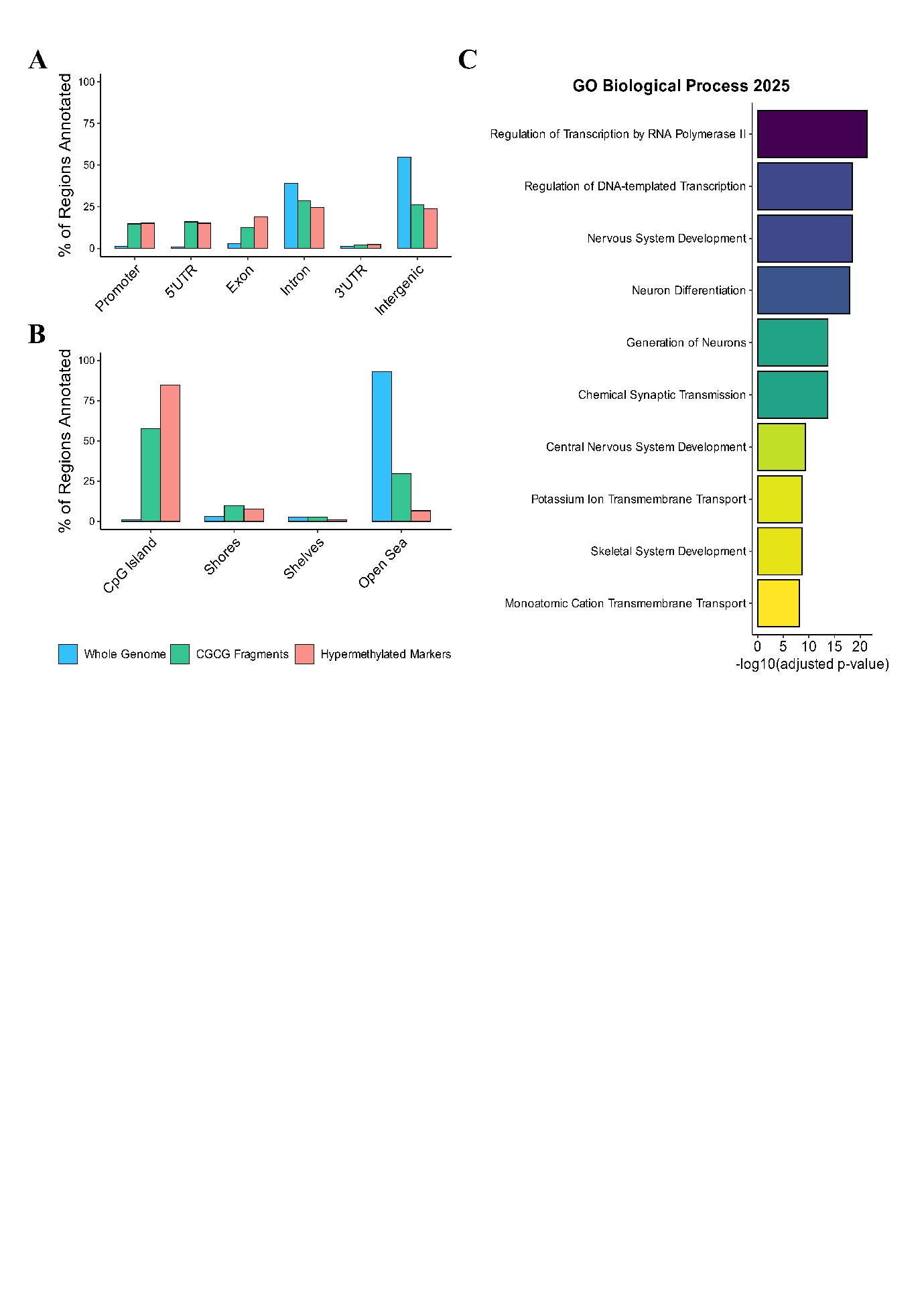
**

**Fig. S10** CPM-normalized read counts for *NPY*, *ITGA4*, and *IRF4* plotted by cancer stage. Spearman correlation between CPM and stage is shown (healthy, *n* = 35; Stage I, *n* = 7; Stage II, *n* = 12; Stage III, *n* = 11; Stage IV, *n* = 17; total, *n* = 82).


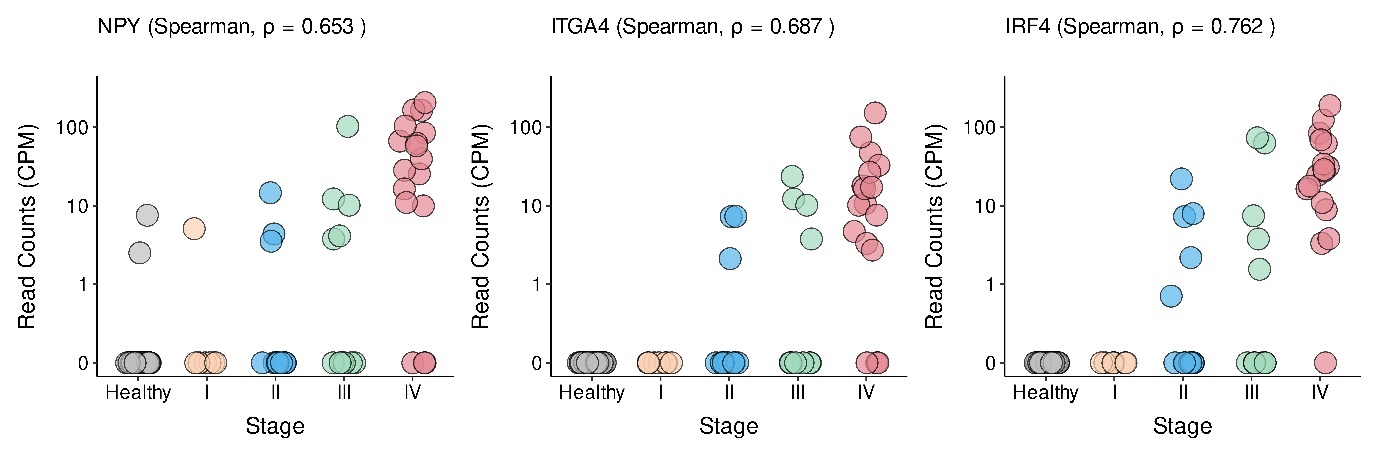


**Fig. S11** Comparison of sequencing and mapping statistics between EM-seq and RECAP-seq triplicates, including average read lengths before and after adapter trimming. GC content, Q30, and average read length were obtained from FASTP, and percent of mapped reads were obtained from Bismark.

| **Method** | **Replicate** | **GC content (%)** | **Q30 (%)** | **Average read length (bp)** | | **Mapped (%)** |
| --- | --- | --- | --- | --- | --- | --- |
|  |  |  |  | **Before adapter trimming** | **After**  **adapter trimming** |  |
| **EM-seq** | Rep1 | 27.7 | 90.9 | 151.0 | 125.0 | 86.0 |
|  | Rep2 | 21.2 | 91.2 | 111.0 | 104.0 | 84.9 |
|  | Rep3 | 21.8 | 91.0 | 111.0 | 103.0 | 84.8 |
| *Average*  *(EM-seq)* |  | 23.6 | 91.0 | 124.3 | 110.7 | 85.2 |
| **RECAP-seq** | Rep1 | 48.6 | 73.6 | 151.0 | 80.5 | 42.2 |
|  | Rep2 | 48.6 | 73.1 | 151.0 | 77.0 | 41.9 |
|  | Rep3 | 48.4 | 72.7 | 151.0 | 80.0 | 43.2 |
| *Average (RECAP-seq)* |  | 48.5 | 73.1 | 151.0 | 79.2 | 42.4 |
